# Correction: Measurements and models of electric fields in the *in vivo* human brain during transcranial electric stimulation

**DOI:** 10.7554/eLife.35178

**Published:** 2018-02-15

**Authors:** Yu Huang, Anli A Liu, Belen Lafon, Daniel Friedman, Michael Dayan, Xiuyuan Wang, Marom Bikson, Werner K Doyle, Orrin Devinsky, Lucas C Parra

Huang Y, Liu AA, Lafon B, Friedman D, Dayan M, Wang X, Bikson M, Doyle WK, Devinsky O, Parra LC. 2017. Measurements and models of electric fields in the *in vivo* human brain during transcranial electric stimulation. *eLife* **6**:e18834. doi: 10.7554/eLife.18834.

Published 07, February 2017

Since publication of the paper, Alex Opitz alerted us of a problem with the calibration of the voltage recordings. When stimulating with sinusoidal currents the Neuroconn stimulator that was used in our experiments specifies stimulation intensity as peak-to-peak amplitude, which we now confirmed by measuring current through a 10kOhm load. We had incorrectly interpreted this as zero-to-peak amplitude. In the first step of analysis we divided all voltage measures by this nominal current value to obtain measures calibrated to 1 mA of stimulation. Due to this misinterpretation, all values we reported in the paper for measured voltages and field magnitudes have to be multiplied by a factor of 2.

In the paper we had concluded that the measured field magnitudes are smaller than predicted by previous computational models. In fact, with this correction there is now remarkable agreement of our results with existing models, as well as *in-vivo* measurements. Our results now match recent reports from monkey and human of approximately 0.8 V/m maximum field amplitudes in the brain when stimulating transcranially with 2 mA current (Opitz et al., 2016; Krause et al., 2017; Kar et al., 2017). After the fitting of conductivities to the corrected voltage measurements we obtain new electric field estimates. These are approximately twice as large as before, though the exact values differ due to the non-linearity of the fitting and prediction process. The estimated fields now match the values obtained with conductivities previously used in the literature. We are correcting here all figures and detailed numerical values in the text as follows (changes are underlined):

In the Abstract:

1. When stimulating at 2 mA, cortical electric fields reach 0.8 V/m, the lower limit of effectiveness in animal studies. When individual whole-head anatomy is considered, the predicted electric field magnitudes correlate with the recorded values in cortical ($\underline{r=0.86}$) and depth ($\underline{r=0.88}$) electrodes.

In the Introduction:

1. For instance, we find that maximal electric field amplitudes are approximately 0.8 V/m when using the generally accepted maximum of 2 mA scalp stimulation.

In the Results:

1. Regardless, the variability observed here sets the lower limit in precision one can expect to $\underline{\pm 0.11}$ mV for 1 mA stimulation (standard deviation pooled across electrodes and subjects; Figure 3C, values for P03). Subsequent recordings were performed at 1 Hz and with stimulation current of 0.25 mA to 1 mA, limited by the dynamic range of the clinical amplifier and patient sensation.

2. For this subject (P03) the measured voltages are tightly correlated across locations with the predicted electric potential values. The same is true for all subjects resulting in high Pearson correlation coefficients, $\underline{r=0.94}\pm 0.04$.

3. As expected, the correlation of predicted and measured electric fields is lower than for the raw potentials (here $\underline{r=0.86}$, $\underline{p=10^{-14}}$, $\underline{N=45}$), as the calculated field is the difference of two close-by measurements, each with some inherent noise. Similar results are obtained for all ten subjects ($\underline{r=0.81\pm 0.09}$), suggesting that the spatial distribution of electric field magnitudes is well predicted by the models. When collapsing all recordings across subjects (Figure 5F and G) we find correlation between measured and predicted field projections of $\underline{r=0.86}$ ($\underline{p=10^{-118}}$, $\underline{N=405}$) and $\underline{r=0.88}$ ($\underline{p=10^{-54}}$, $\underline{N=164}$) for cortical and depth electrodes respectively. Note also that the measured field projections in depth electrodes are nearly as strong as in cortical electrodes (Figure 5F vs. G; standard deviation of measured field projections across electrodes are $\underline{0.059}$ V/m and $\underline{0.065}$ V/m, respectively).

**Figure 2.**
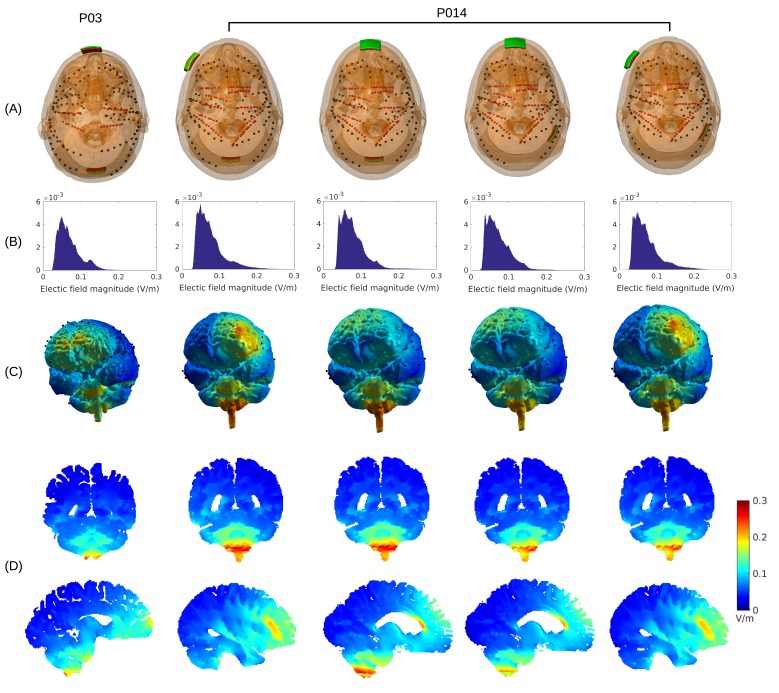


**Figure 3.**
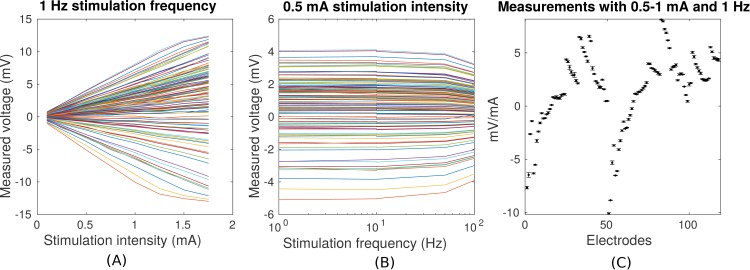


4. Maximal field intensities for the mid-line configuration (Figure 1) are 0.28 V/m ± 0.06 V/m across the ten subjects (Figure 6A). For an intensity of 2 mA typically used in clinical trials this would result in fields of 0.56 V/m. For other stimulation configurations on Subject P014 with electrodes away from the mid-line (Figure 2A, which is more typical for clinical interventions), maximum intensity on cortex for 1 mA stimulation was 0.25 V/m and 0.10 V/m at the fronto-lateral and lateral occipital locations respectively. We also model other electrode configurations commonly used clinical trials, e.g., M1–SO (Figure 6A). For the three configurations tested here, maximum field intensities in cortical locations was 0.38 V/m ± 0.09 V/m, again for 1 mA. To provide a sense of what intensities are reached in more extended areas (not just at the 1 mm^3^ voxel with largest value) Figure 6A also reports the 95th percentile (0.14 V/m ± 0.02 V/m)…

5. For a given predicted electric field intensity, measurements vary by approximately 0.05 V/m. … Assuming that prediction accuracy scales linearly with predicted electric field intensity, this suggests that prediction error for maximal field intensities is in the order of 0.10 V/m.

6. The maximal values at a given depth summarized for all subjects and montages indicate that deep brain areas may experience electric fields that are comparable in magnitude to the cortical surface (Figure 6C, e.g. approximately midway between the center of the brain and the cortical surface (normalized distance 0.25) field magnitude were $\underline{0.21\pm 0.04}$ V/m for these 10 subjects with the mid-line montage).

7. First, conductivity values reported in the literature used in existing models appear to give similar estimate of the electric field magnitudes (see values in ’Conductivity optimization’).

**Figure 5.**
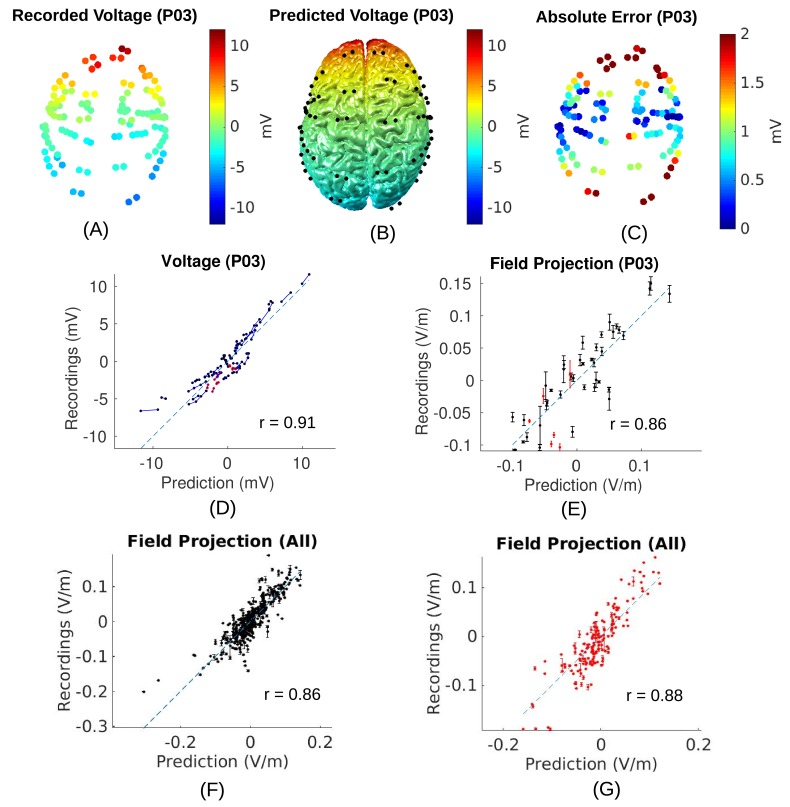


**Figure 6.**
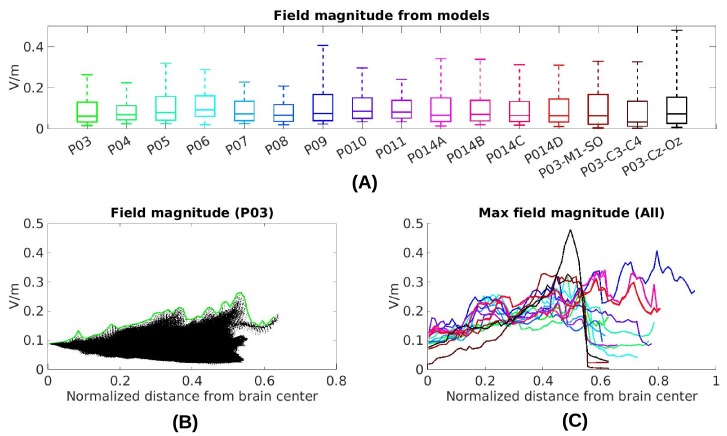


8. … measured electric field magnitudes in subject P03 were close to the predicted values, as indicated by the slope $\underline{s=0.72}$ of the best linear fit of the measured versus predicted field projections shown in Figure 7B. This is true for all subjects (for voltage: $\underline{s=0.83\pm 0.24}$; for field: $\underline{s=0.68\pm 0.21}$. Figure 8C and D under “literature”). The conductivity values typically used in the literature appear to correctly estimate the amount of current that crosses the skull and the amount shunted through the scalp.

9. The median of the optimal conductivities are 0.03 ± 0.01 S/m for bone, 0.29 ± 0.10 S/m for skin, 0.38 ± 0.05 S/m for white matter and 0.82 S/m for gray matter (Figure 8E–G; ± indicates the median of the estimation uncertainty across subjects). These differ from the literature values (bone: 0.01 S/m; skin: 0.465 S/m; white: 0.126 S/m; gray: 0.276 S/m), but are largely in the same proportions.

10. Compared to models using literature conductivities, the models with median values across subjects give significantly better accuracy in terms of predicting the electric field distribution (pairwise t-test: $\underline{t(12)=2.36}$, $\underline{p=0.04}$), Figure 8B, but the magnitude is not significantly different ($\underline{t(12)=1.51}$, $\underline{p=0.16}$).

**Figure 7.**
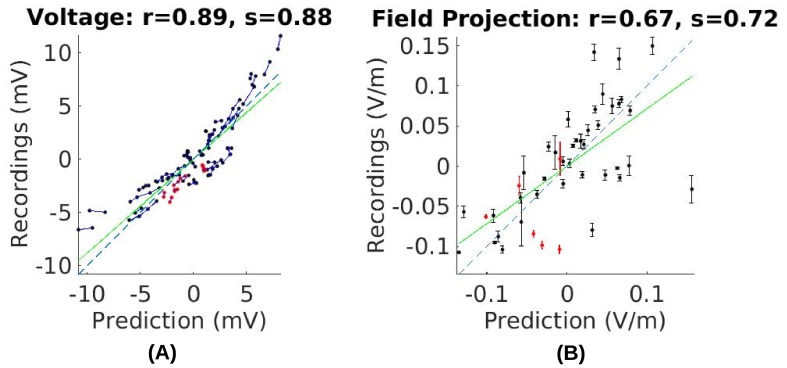
Comparison of recorded values with model predictions using literature conductivity values for Subject P03 scaled to 1 mA. Points falling on the dashed blue line represent perfect prediction (slope s=1). The literature values gives close estimates of electric field magnitude (measurements are 72% of predicted values, $\underline{s=0.72}$, green line). Skin, skull and brain conductivities are optimized to minimize prediction error for field projections (i.e. minimize mean square distance from dashed line in panel (B)) which corrects this magnitude mismatch, and is shown in Figure 5E.

11. Since the MRI of most patients is truncated at the base of the skull, we extended the field of view (FOV) using a standard head that captures the average anatomy of the lower head (see ’General procedure’). This extended FOV gives significantly better predictions as compared to the original FOV, in terms of the electric field magnitudes ($\underline{t(10)=6.17}$, $\underline{p=10^{-4}}$) …

12. We next tested the importance of CSF by removing the CSF compartment from the intact model … This did … deteriorate the accuracy in predicting the magnitude of electric fields (comparing slopes, $\underline{t(12)=4.39}$, $\underline{p=10^{-4}}$). Incorporating heterogeneous conductivities for various bone compartments does not appear to provide a reliable improvement on magnitude estimates (RM vs. RM+3skull: $\underline{t(12)=1.72}$, $\underline{p=0.11}$) …

In the Discussion:

1. Our main finding is that the intensities of electric field reach 0.8 V/m when using 2 mA transcranially. This is close to previous predictions using computational models (Datta et al., 2009).

2. We find that individualized models provide predictions of the spatial distribution of currents with an accuracy of $\underline{r=0.86}$ for cortical electrodes and $\underline{r=0.88}$ for depth electrodes when pooling data across all subjects.

**Figure 8.**
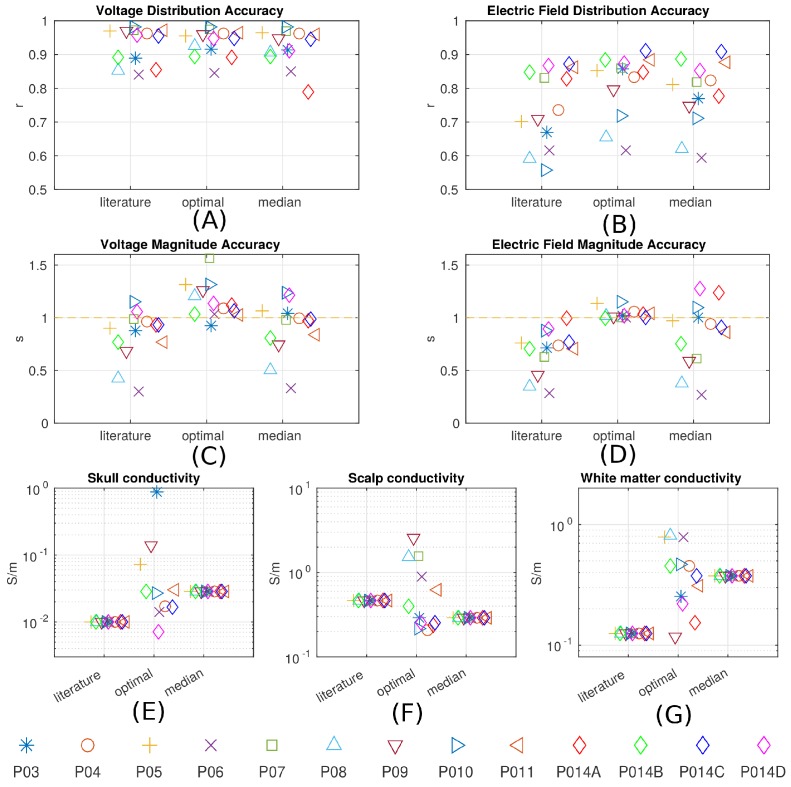


3. Electric field magnitudes in cortex can be as high as 0.4 V/m for 1 mA stimulation current. For typical electrode configurations used in clinical trials maximal field intensity can reach 0.8 V/m when applying 2 mA. More extended areas can reach a value of 0.28 V/m (95th percentile) under 2 mA stimulation. For some electrode montages, current is shunted into deeper areas through highly conductive CSF with maximal values reaching 0.21 V/m. The present data is broadly in agreement with previous modeling estimates with maximal intensities around 1 V/m (e.g., Datta et al., 2009) and 95th percentile of 0.35 V/m (e.g., Datta et al., 2012).

4. A recent study has reported the electric field produced by TES as measured by stereotactic EEG electrodes in two epilepsy surgical patients (Opitz et al., 2016). While the study does not leverage or evaluate accuracy of computational models, it does estimate maximal electric field to be 0.5 V/m with 1 mA TES. This closely approximates the intensity estimated from our current data (0.4 V/m for 1 mA stimulation). These results are in remarkably close agreement if one considers the differing placement of the stimulation electrodes.

5. After calibrating the models the overall magnitudes of electric field were predicted with a single set of parameters across all subjects without a bias ($\underline{s=0.84\pm 0.31}$). … The individualized models at 1 mm${}^{3}$ resolution provide predictions that correlated with measured electric field intensities ($\underline{r=0.81\pm 0.09}$) across the ten subjects (Figure 8B). … when using a single set of values (median) we find similar performance ($\underline{r=0.78\pm 0.10}$).

6. The overall correlation of predicted and measured fields is 0.86 for cortical electrodes, and 0.88 for depth electrodes (Figure 5F/G).

7. We found relatively little variation in the voltage measurements (±0.11 mV, e.g., Figure 3C), and note that the variations we did observe were likely due to subject movement.

8. We conclude that with ($\underline{r=0.78\pm 0.10}$) the spatial distribution of electric field predicted by computational models is likely not far from the actual field distributions when taking into account their inherent variability across time, due to subject motion, position, blood pressure, electrode contact, etc.

In the Methods

1. The stimulation protocol used the Neuroconn DC Stimulator Plus (NeuroConn, Germany), with a sinusoidal waveform, at variable frequencies and intensities between 0.25 mA and 1 mA, zero-to-peak amplitudes.

In closing we note that all new analyses and figures exclude duplicate measurement points following the considerations discussed in an earlier commentary. We also adjusted electrode location in a few subjects based on more detailed description from AAL who placed electrodes underneath the bandage. All corrections are also reflected in the data repository http://dx.doi.org/10.6080/K0XW4GQ1.
