# Supplementary material for: Measurements and models of electric fields in the in vivo human brain during transcranial electric stimulation
Source: eLife. 2017 Feb 7;6:e18834. doi: 10.7554/eLife.18834 (PMC5370189; doi:10.7554/eLife.18834)
Supplement: Figure 5—source data 1. — The predicted values are from the individually optimized models. DOI: http://dx.doi.org/10.7554/eLife.18834.007 [file elife-18834-fig5-data1.ppt]

## Slide 1
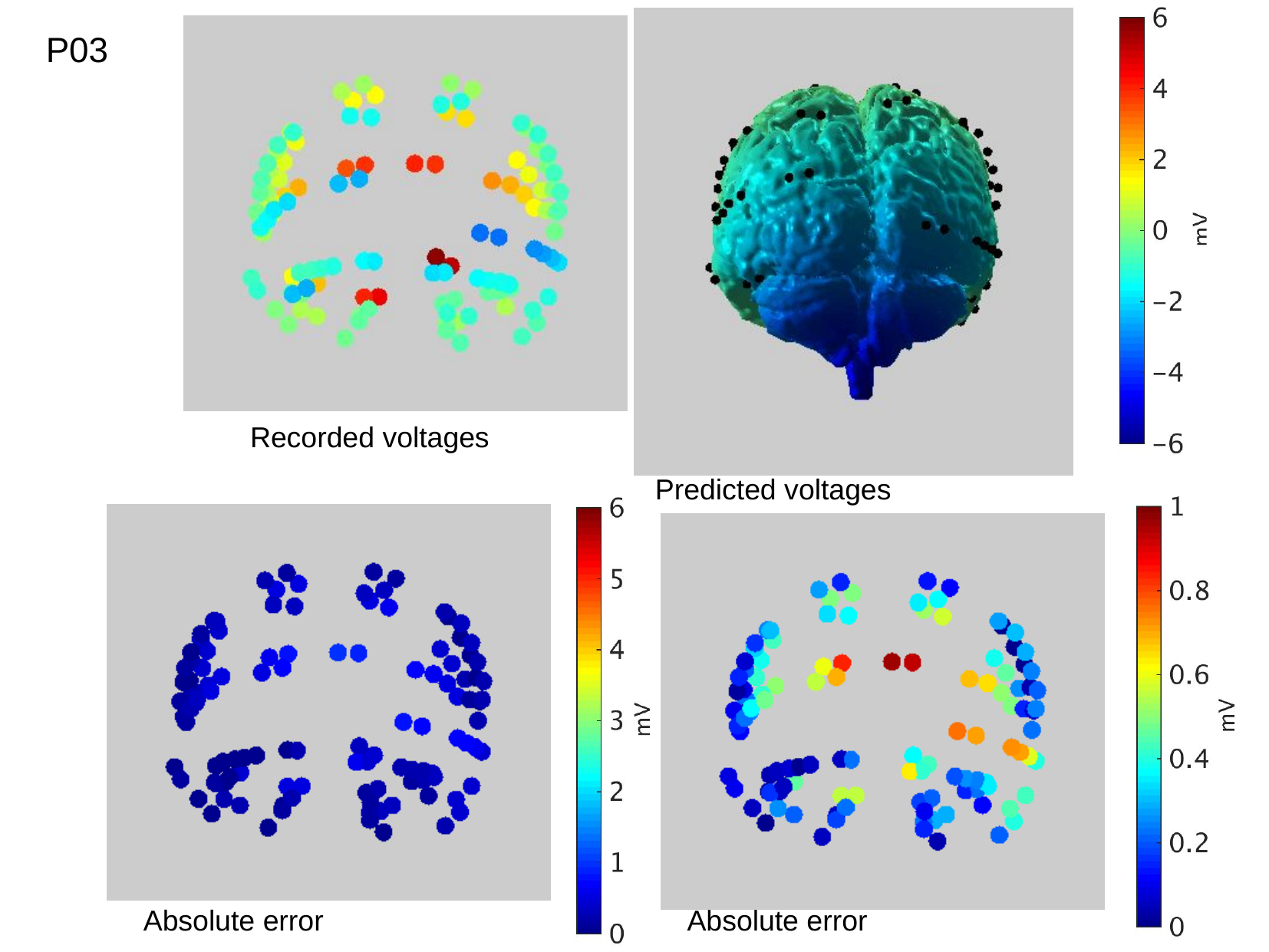

P03
Recorded voltages
Predicted voltages
Absolute error
Absolute error

## Slide 2
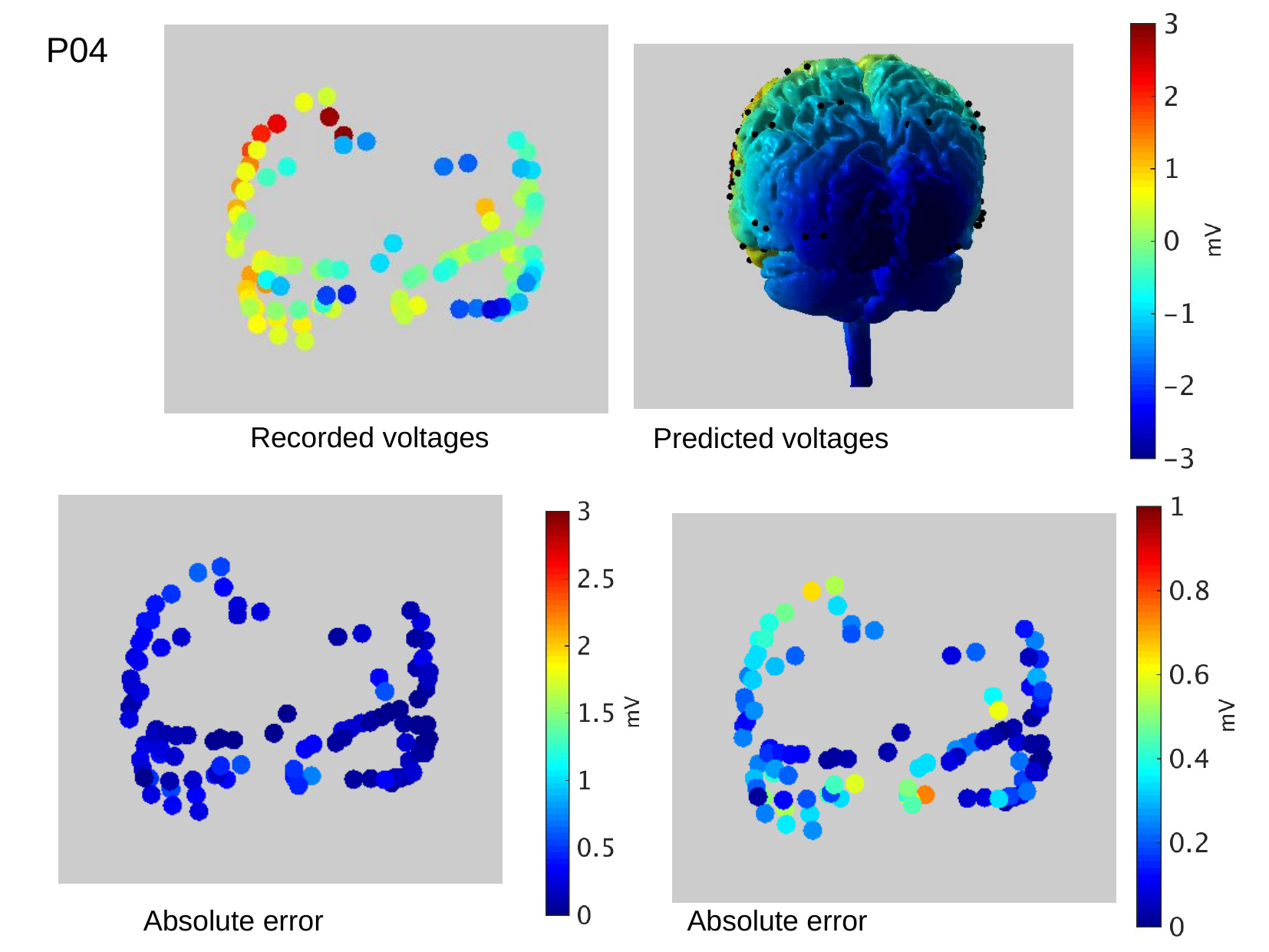

P04
Recorded voltages
Predicted voltages
Absolute error
Absolute error

## Slide 3
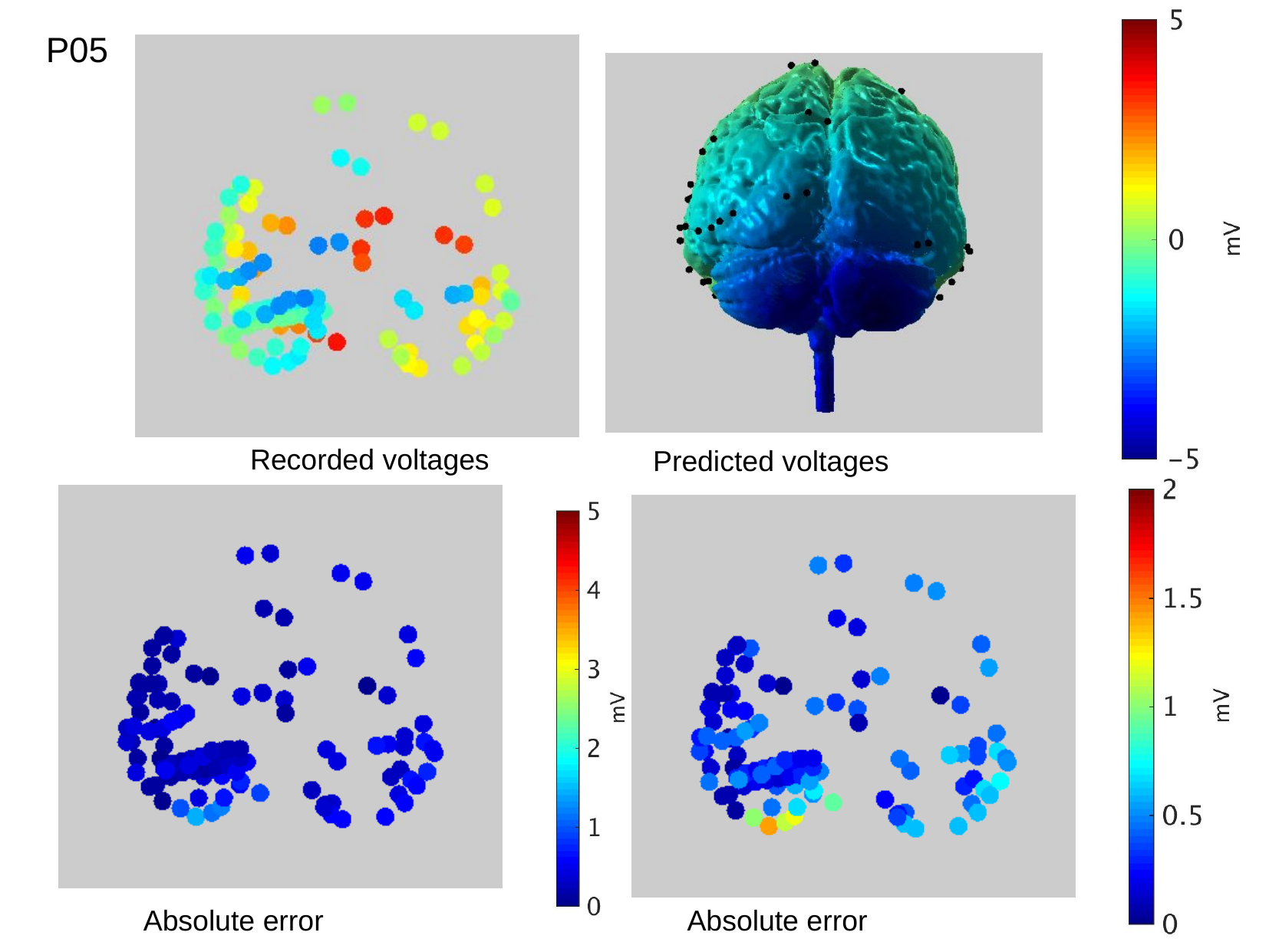

P05
Recorded voltages
Predicted voltages
Absolute error
Absolute error

## Slide 4
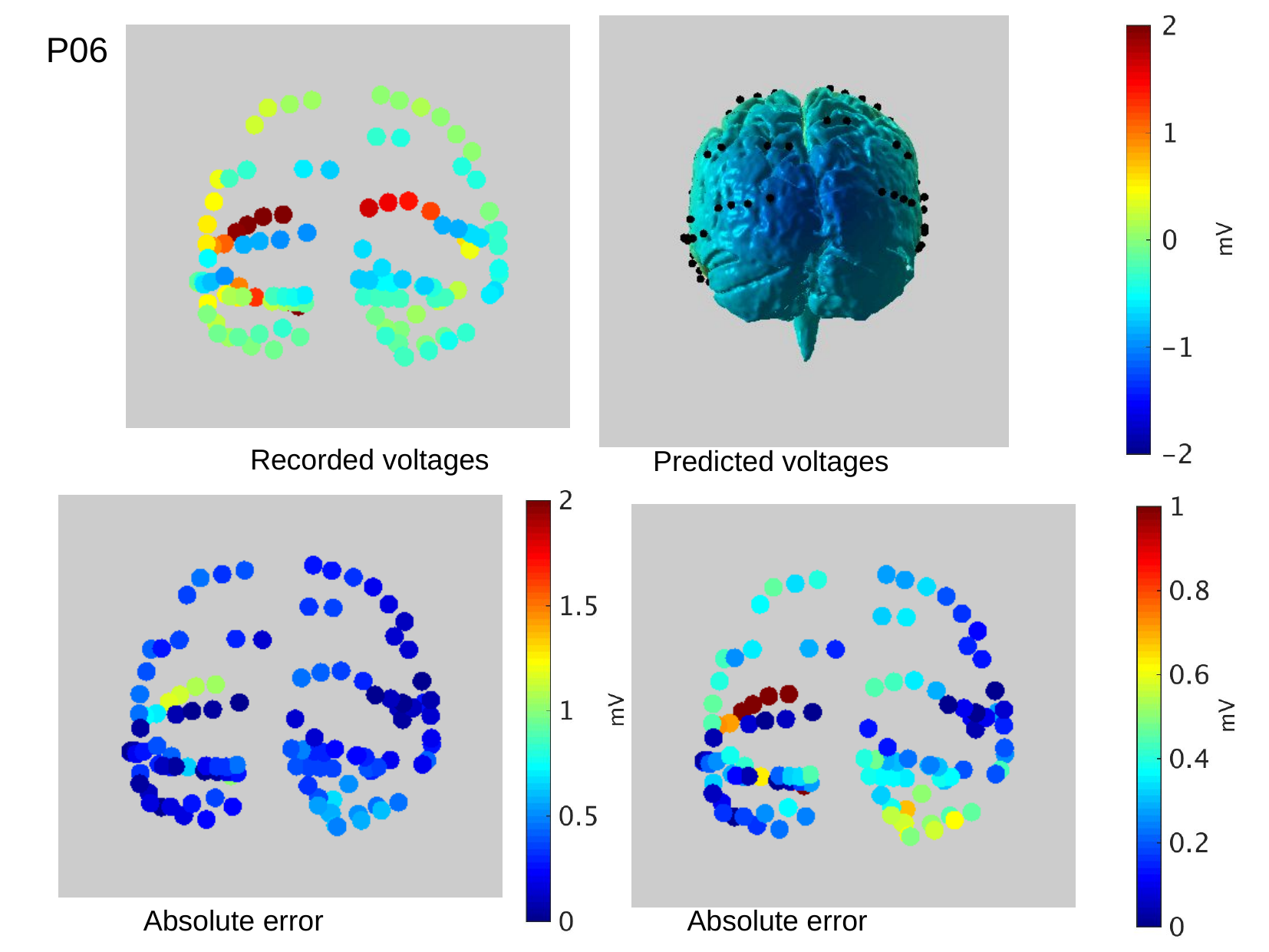

P06
Recorded voltages
Predicted voltages
Absolute error
Absolute error

## Slide 5
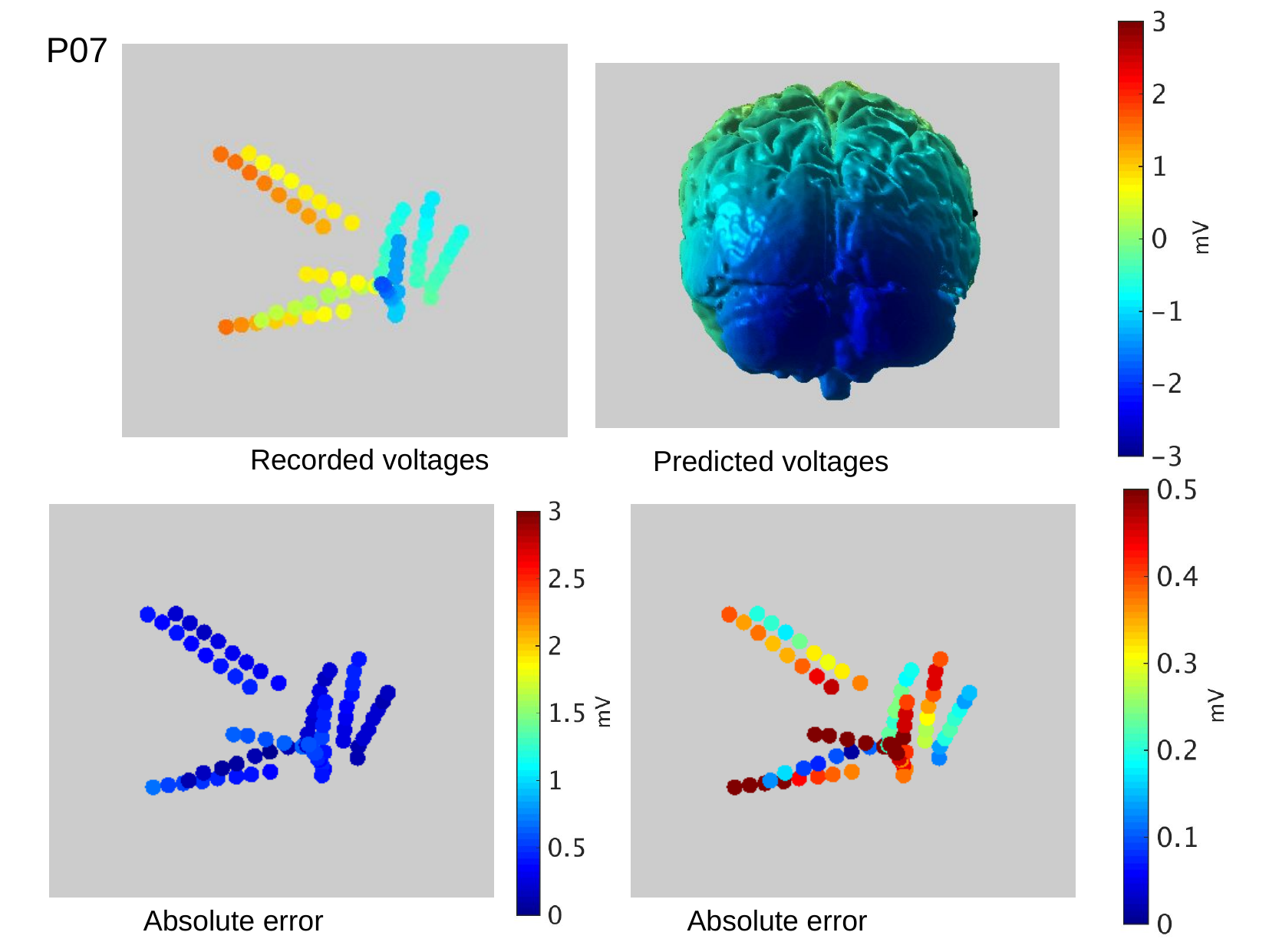

P07
Recorded voltages
Predicted voltages
Absolute error
Absolute error

## Slide 6
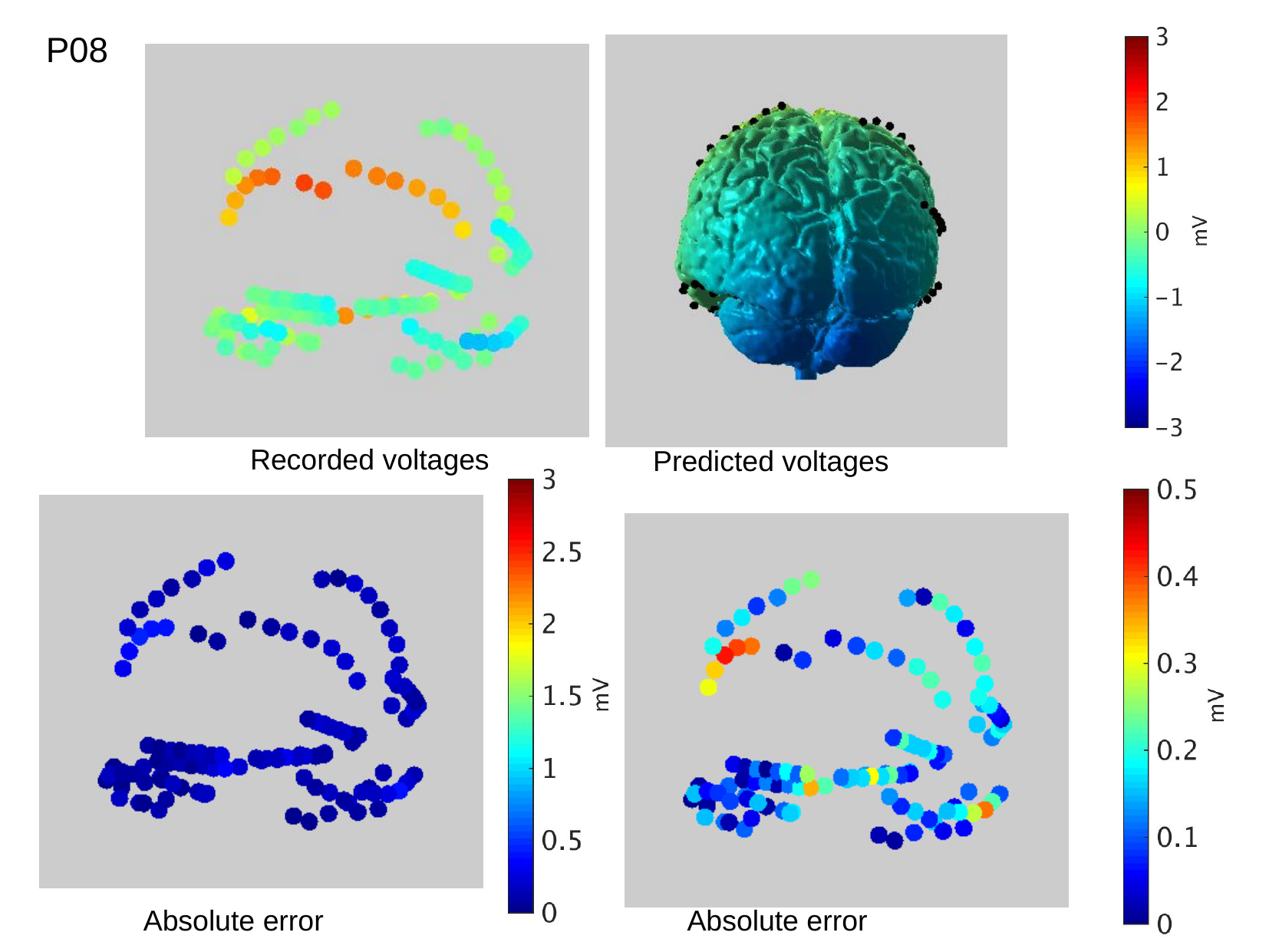

P08
Recorded voltages
Predicted voltages
Absolute error
Absolute error

## Slide 7
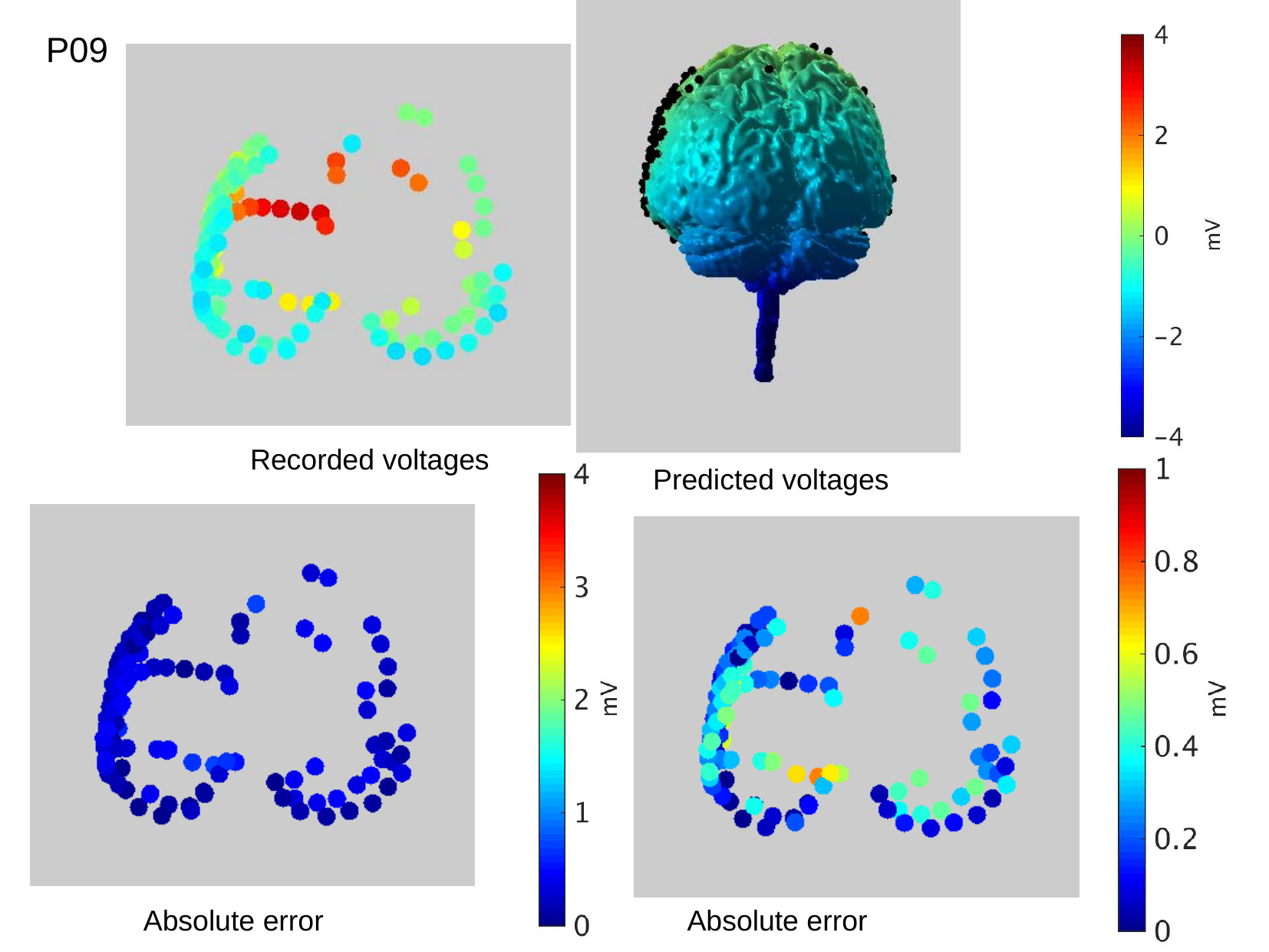

P09
Recorded voltages
Predicted voltages
Absolute error
Absolute error

## Slide 8
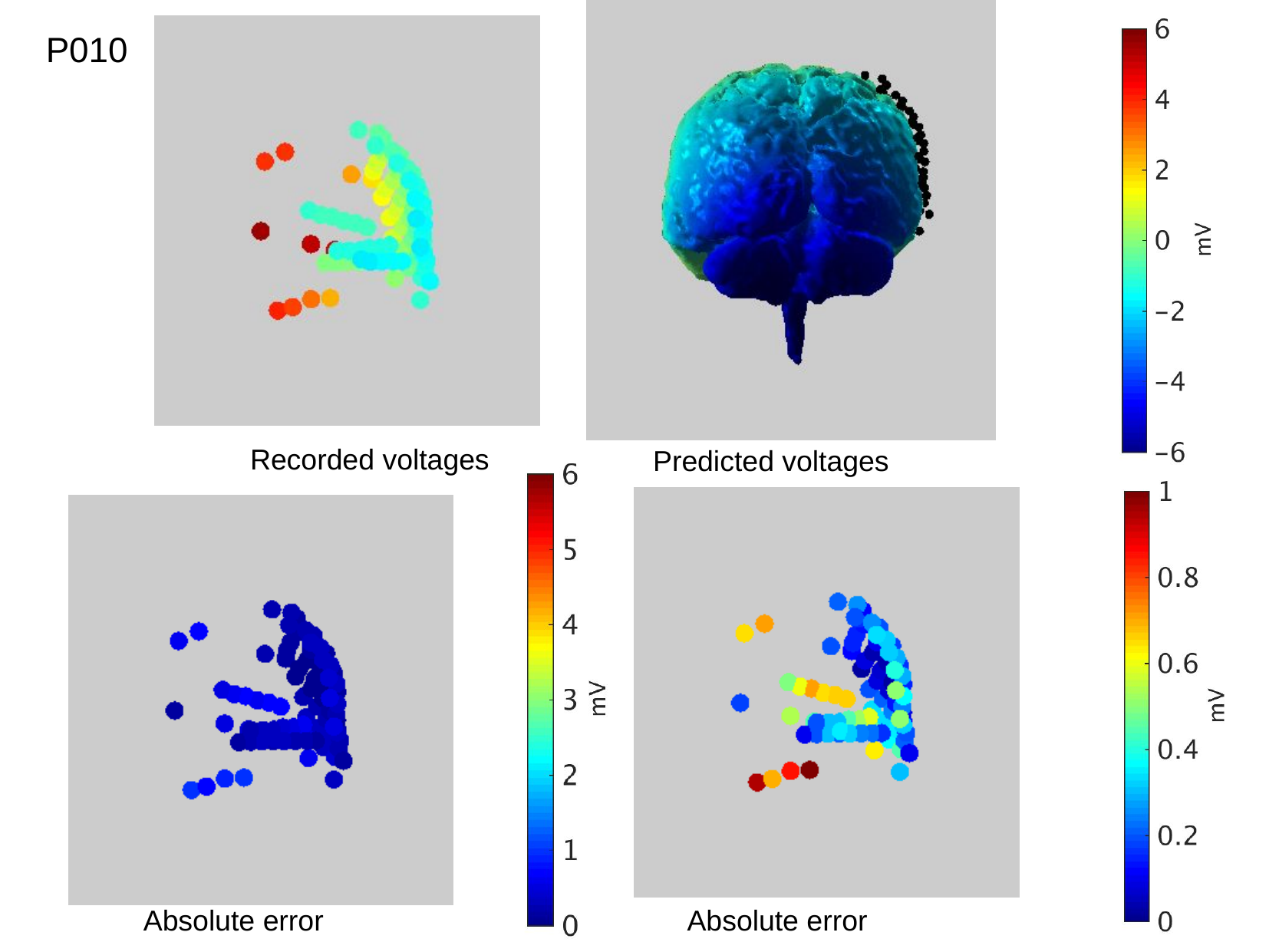

P010
Recorded voltages
Predicted voltages
Absolute error
Absolute error

## Slide 9
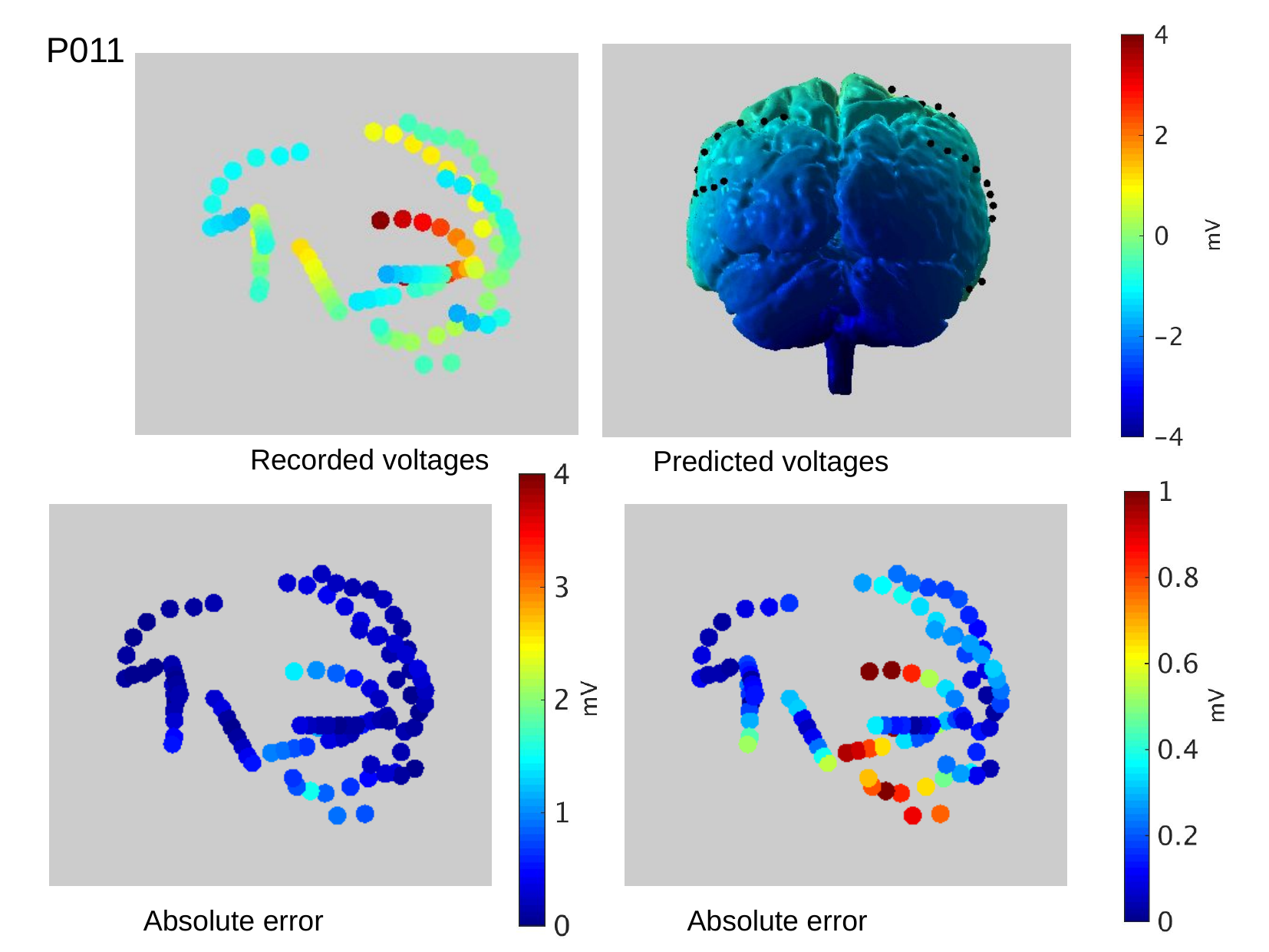

P011
Recorded voltages
Predicted voltages
Absolute error
Absolute error

## Slide 10
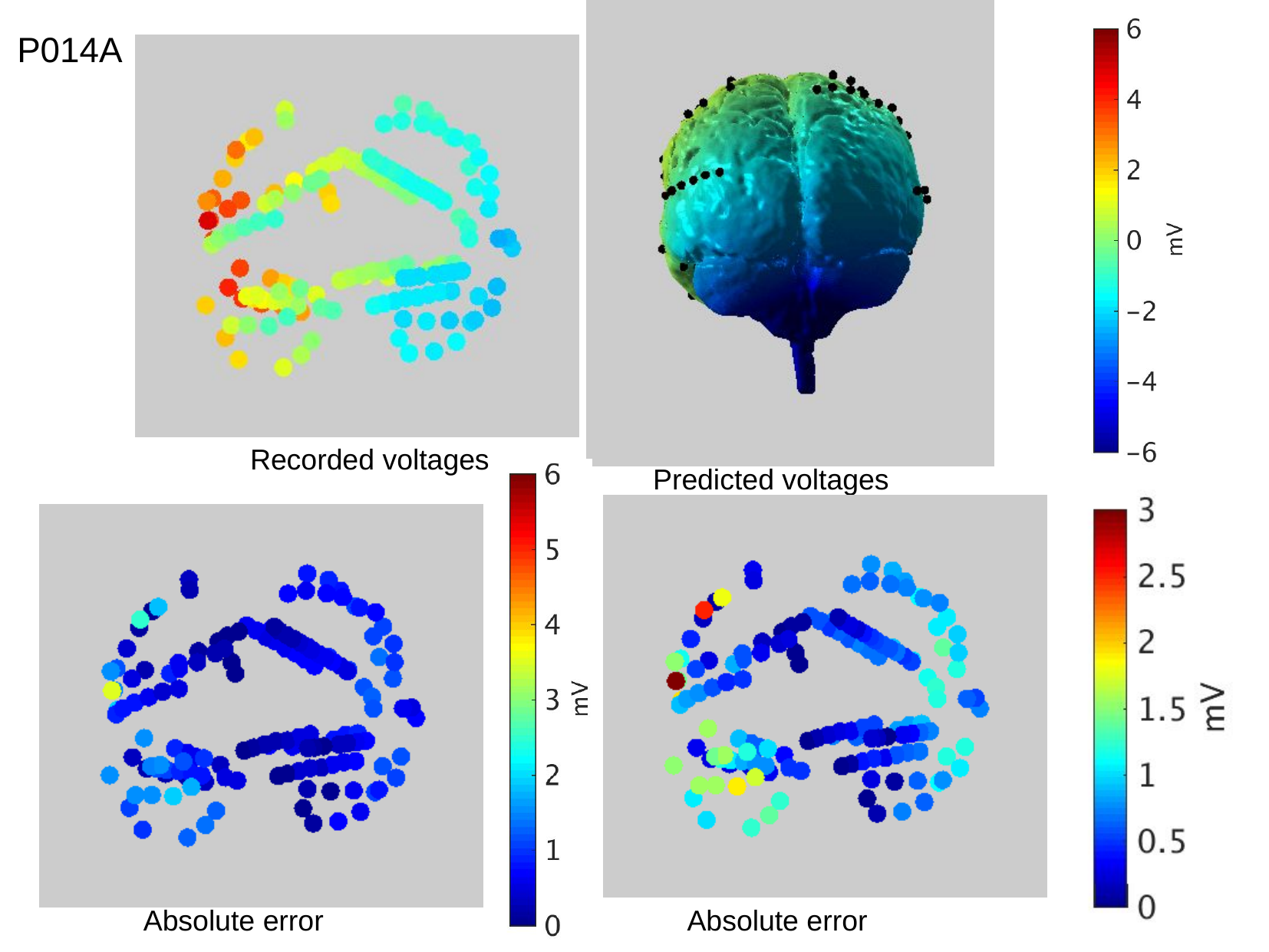

P014A
Recorded voltages
Predicted voltages
Absolute error
Absolute error

## Slide 11
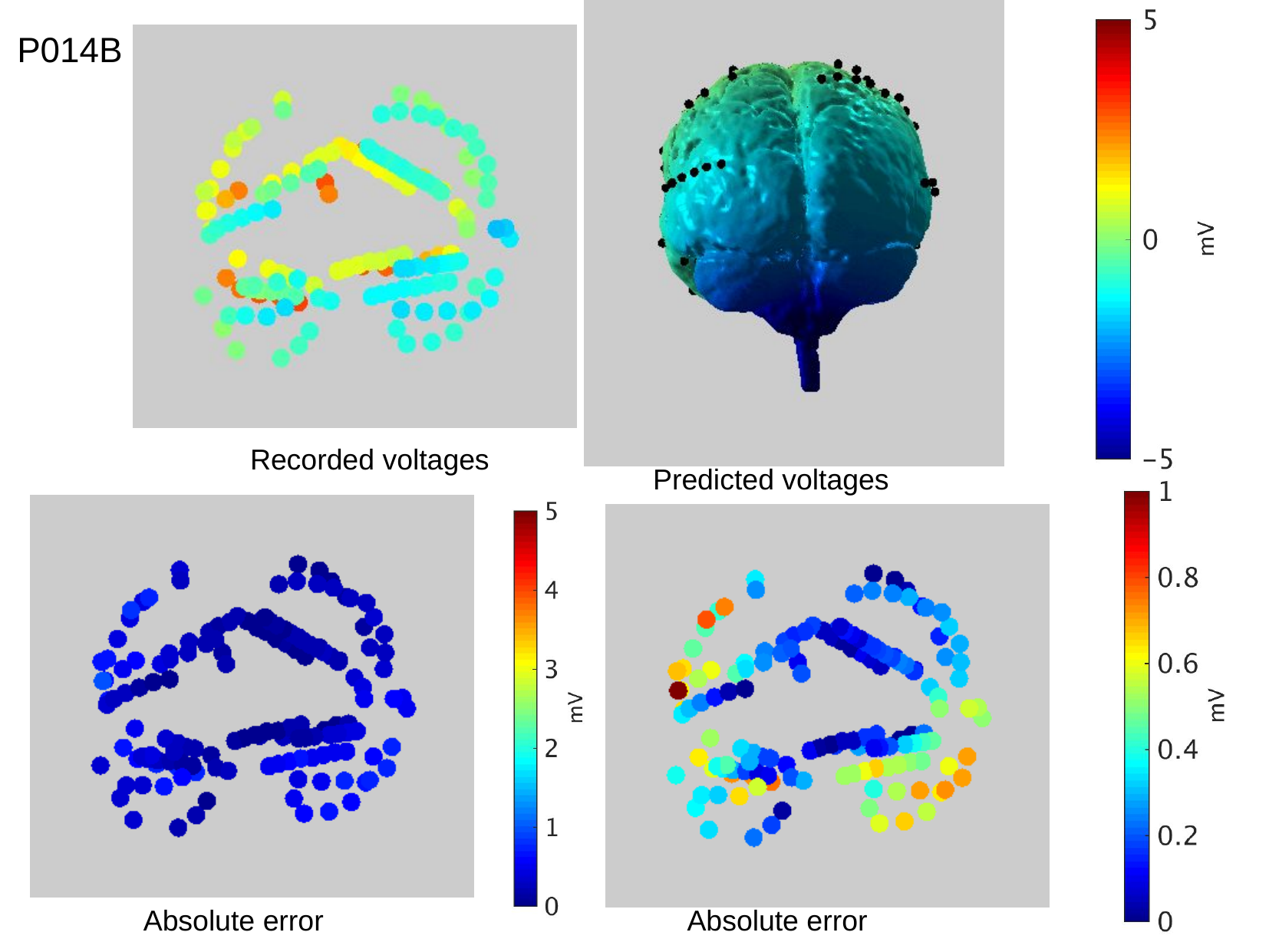

P014B
Recorded voltages
Predicted voltages
Absolute error
Absolute error

## Slide 12
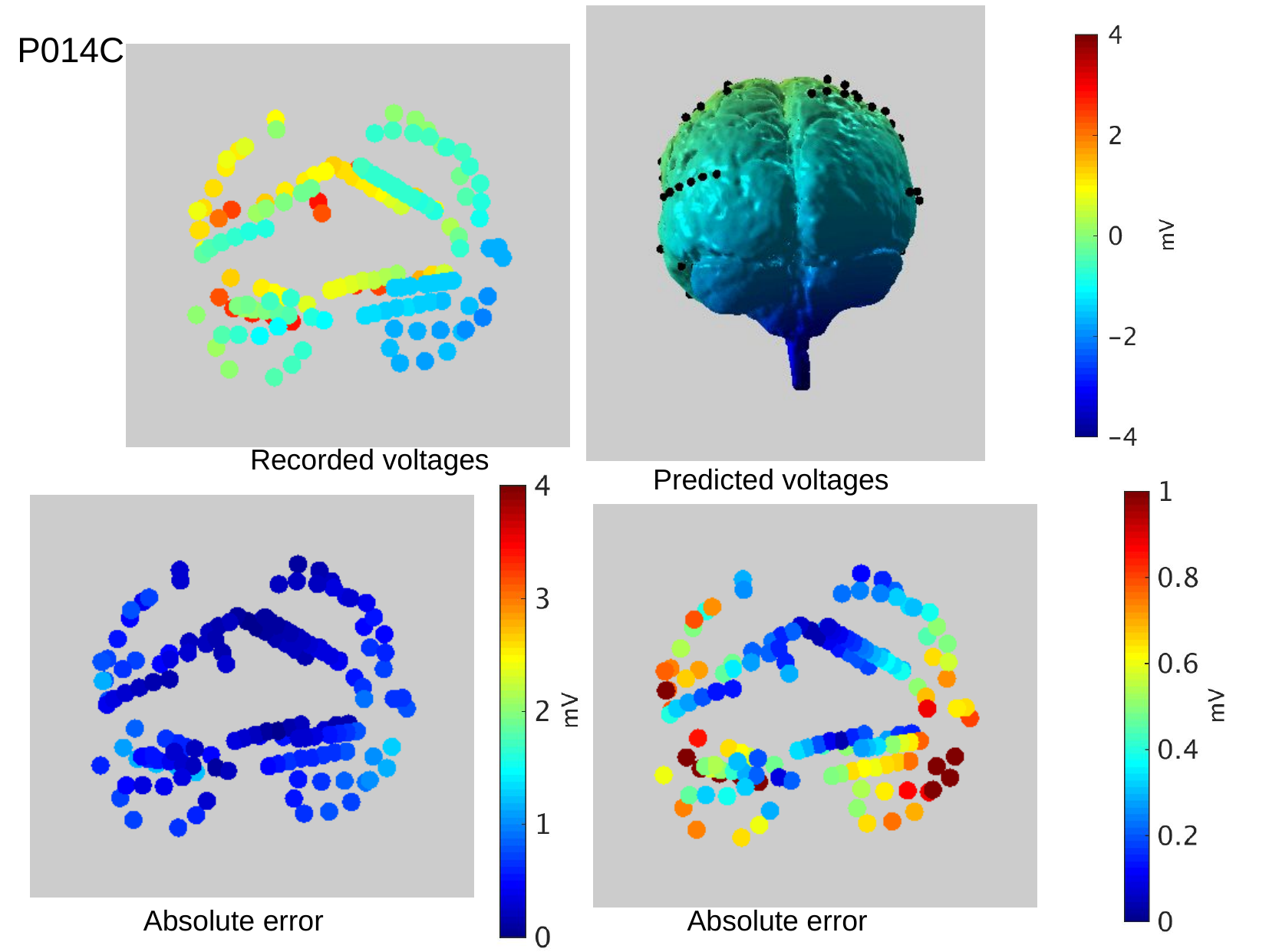

P014C
Recorded voltages
Predicted voltages
Absolute error
Absolute error

## Slide 13
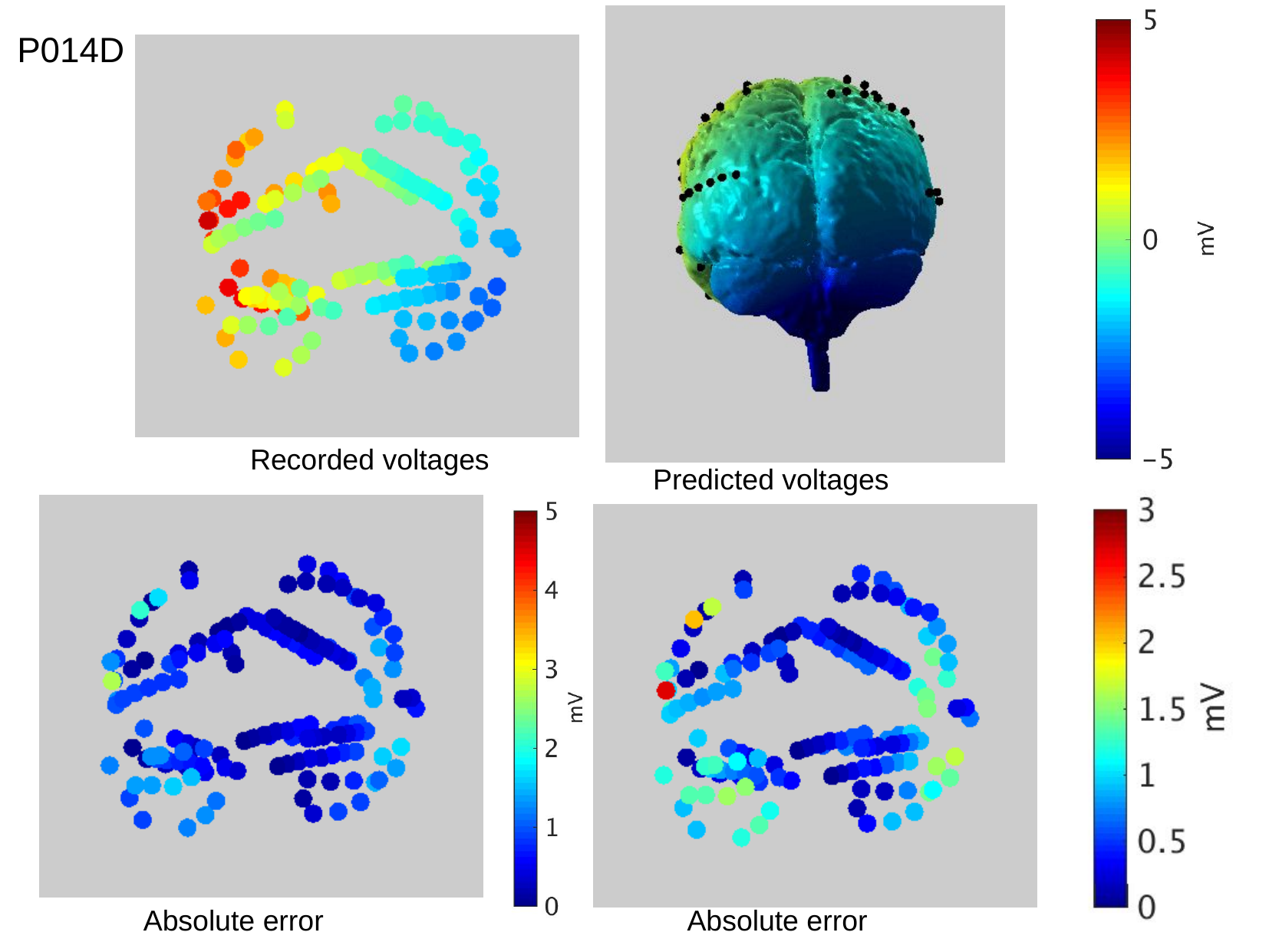

P014D
Recorded voltages
Predicted voltages
Absolute error
Absolute error
